# The importance of health advocacy in Canadian postgraduate medical education: current attitudes and issues

**Published:** 2015-12-11

**Authors:** Alexander Poulton, Heather Rose

**Affiliations:** 1 Faculty of Medicine, Dalhousie University; 2 Department of Pediatrics, Dalhousie University

## Abstract

**Background:**

Health advocacy is currently a key component of medical education in North America. In Canada, Health Advocate is one of the seven roles included in the Royal College of Physicians and Surgeons of Canada’s CanMEDS competency framework.

**Method:**

A literature search was undertaken to determine the current state of health advocacy in Canadian postgraduate medical education and to identify issues facing educators and learners with regards to health advocacy training.

**Results:**

The literature revealed that the Health Advocate role is considered among the least relevant to clinical practice by educators and learners and among the most challenging to teach and assess. Furthermore learners feel their educational needs are not being met in this area. A number of key barriers affecting health advocacy education were identified including limited published material on the subject, lack of clarity within the role, insufficient explicit role modeling in practice, and lack of a gold standard for assessment. Health advocacy is defined and its importance to medical practice is highlighted, using pediatric emergency medicine as an example.

**Conclusions:**

Increased published literature and awareness of the role, along with integration of the new 2015 CanMEDS framework, are important going forward to address concerns regarding the quality of postgraduate health advocacy education in Canada.

## Introduction

The notion that physicians must act as health advocates is one that is deeply woven into the fabric of medical education and accreditation. In the United States, the Accreditation Council for Graduate Medical Education (ACGME) has the role of patient advocate built into one of the six core competencies within the ACGME Common Program Requirements.^1^ In Canada, health advocacy is integral to a number of key frameworks for physician competence including the *Four Principles* of the College of Family Physicians of Canada (CFPC), the Canadian Medical Association Code of Ethics, and the Canadian Association of Emergency Physicians (CAEP) Mission Statement.^2^–^4^ Most notably, in terms of training and education, it is one of the seven roles within the Canadian Medical Education Direction for Specialists (CanMEDS) framework employed by the Royal College of Physicians and Surgeons of Canada (RCPSC).^5^

The RCPSC is the body responsible for standards of accreditation, training, and examination in residency. Building off of the Educating Future Physicians for Ontario (EFPO) project they developed a foundation for physician competency known as the CanMEDS competency framework.^1^,^6^ This framework, adopted in 1996 and subsequently revised, surrounded seven roles of the specialist physician: Medical Expert, Communicator, Collaborator, Manager, Professional, Scholar, and Health Advocate.^7^ These roles constitute the competencies deemed necessary to meet the needs of patients, communities and populations served by physicians. The roles have not all been seamlessly integrated into practice, however, with the Health Advocate role providing a particular challenge.^6^,^7^ This paper highlights the importance of the Health Advocate role in current practice, utilizing pediatric emergency medicine as an example, and describes current attitudes and barriers affecting health advocacy education.

## Methods

A literature review was undertaken to elicit research surrounding health advocacy in Canadian postgraduate medical education. A PubMed search strategy was used which included a mix of index terms and keywords. The terms “CanMEDS,” “medical competency,” or “postgraduate medical education” were used along with equivalents such as “CanMEDS based” and related index terms including “education, medicine, graduate.” This was overlapped with a further inquiry for articles using the search terms “advocacy” or “health advocacy” along with related keyword terms including “health advocate” and index terms including “patient advocacy.” No parameters were placed on language of publication or date of publication in order to maximize the amount of literature available. The preliminary search yielded 185 articles, the titles/abstracts of which were reviewed directly by the author for relevance to the purpose of this review. Articles discussing the utility of the CanMEDS roles in practice outside of Canada were excluded. Fifteen articles reflective of the goals of this review were included then mined directly for related research. The PubMed “related citation” function was also utilized along with an in-site search of the RCPSC website and a Google Scholar search.

### Definition of health advocacy

A number of general definitions of health advocacy exist in the literature. Advocacy has previously been defined as pleading the cause of those who require assistance or are unable to speak for themselves.^8^ This can be viewed as a paternalistic or prescriptive approach to advocacy.^6^ It has also been described as primarily a team enterprise in which physicians work collectively with patients and others to achieve common health goals, an approach which can be seen as empowering or facilitative.^6^,^9^ These broad types of health advocacy can occur directly, at the individual patient care level, or indirectly at the community or population level. Importantly to education, health advocacy has been defined as more than simply an idea. Rather, advocacy has been conceptualized as a skill requiring teaching and practice.^10^

The important position ascribed to health advocacy in health policy, makes it imperative that a strong working definition be in place, beyond the general conceptions. The 2005 CanMEDS framework provides a specific definition stating, “As *Health Advocates*, physicians responsibly use their expertise and influence to advance the health and well-being of individual patients, communities, and populations.”^5^ The role of Health Advocate includes four key competencies:

Physicians are able to:

1. Respond to individual needs and issues as part of patient care;
2. Respond to the health needs of the communities that they serve;
3. Identify the determinants of health of the populations that they serve;
4. Promote the health of individual patients, communities, and populations.

More recently, the landscape of CanMEDS health advocacy has begun to evolve from this, with a movement towards the new 2015 CanMEDS template.^11^ The revision has seen a shift from four to two key competencies within health advocacy, addressing (a) the individual patient’s needs within and beyond the clinical setting, and (b) the needs of the communities and populations they serve through system level change in a “socially accountable manner.”^11^ Critically, the newer formulation has increased the emphasis on advocating *with* patients and larger groups, as opposed to *for* them. This potentially addresses concerns that the role was previously seen as overwhelming to physicians, and perhaps too heavily reflective of the prescriptive concept of advocating on behalf of others.^9^

Through the lens of both the more generalized concepts of health advocacy and the specific CanMEDS concepts of health advocacy, it is possible to begin to appreciate the Health Advocate role in various fields of medicine, with pediatric emergency medicine providing a strong example.

### Role in pediatric emergency medicine

In some contexts more than others, health advocacy is at the forefront of care. This is particularly evident when working with patients who are not fully capable or knowledgeable enough to advocate for themselves. One specific demographic that requires this magnitude of advocacy at times is the pediatric population. By definition, pediatric patients require those in positions of authority to make decisions and deliberate on their behalf to improve their health, including family members, caregivers and physicians. The vulnerability of their state creates an increased need for advocacy in these patients as much in times of disease as in times of health, necessitating health advocacy both directly at the patient level and indirectly at the community level. At the same time, the caregiver aspect to pediatric medical presentations creates a further area for direct health advocacy; addressing the wellbeing of the parent or guardian by working with them to assist with their needs.

In the emergency department (ED), physicians occupy a unique position in terms of their potential for health advocacy based on multiple factors. Many patients utilize the ED as their only source of health care intervention, so emergency physicians may be the only clinicians who can advocate for these patients. These physicians have a wide scope of interaction with other health care professionals. ED presentations are often instances when patients are receptive to health promotion during “teachable moments” where topics including injury prevention can be reinforced.^12^ More broadly, emergency room physicians have historically been very successful at advocating through organizations and at the community level.^13^

Taking ED and pediatric practice together, we see that health advocacy, as a physician role, should be considered central to the practice of pediatric emergency care. The vulnerability of the pediatric patient population combined with the advocacy potential of emergency room physicians converges in the pediatric ED where physicians are in a particularly strong position to advocate for patients. While the need for health advocacy in this domain is highly visible, the same applies to some extent to all other medical specialties. Physicians are experts in their given field, are committed to the health of patients, and have considerable influence so they should be utilizing that knowledge and influence to assist their patients in meeting their health care goals and to promote the health of the community they serve.

### Attitudes towards health advocacy

Despite the significant advocate role physicians should play and have been asked to play, there is a paucity of literature related to education in health advocacy in Canadian postgraduate medical training.^7^,^14^,^15^ This lack of literature and evidence in and of itself is an issue which must be addressed to promote improved health advocacy education. Current literature does, however, provide some insight into the current state of health advocacy in Canadian medical education.

Health advocacy, when compared to other CanMEDs roles, is considered to be one of the least relevant roles by residents and attending physicians.^7^,^14^,^16^ When discussed in isolation, however, health advocacy was found to be important to medical education and practice in surveys of urology residents, obstetrics and gynecology residents, and recent general internal medicine graduates, respectively.^17^–^19^ In each case there was a gap noted between health advocacy being listed as a valuable competency and the learners’ perception that their advocacy training needs were not being adequately met.^17^–^19^ The implication may be that given conflicting priorities in postgraduate education, programs are devaluing health advocacy education relative to other competencies, with the result that solid education in the field is not being provided. A survey of dermatology residents echoed these findings as the majority of respondents felt they were not adequately prepared for the Health Advocate role.^20^ One older study of pediatric graduates certified between 1999 and 2003 reported dissenting findings as the majority of respondents felt adequately prepared for the role of Health Advocate following their training.^21^

With regards to educators in emergency medicine, which is of particular interest due to the potential for advocacy, Bandiera and colleagues found that faculty who participated in a series of focus groups felt that advocacy beyond the direct patient encounter level was not relevant or easy to teach during ED rotations.^14^ This finding was echoed by Dobson and colleagues, where practicing physicians with a particular interest in advocacy felt that advocacy beyond the direct patient level was challenging to conceptualize and enact.^22^ Another study of academic emergency physicians showed health advocacy was not a priority for professional development.^23^ This reduced priority is of paramount importance as it sheds some light on the issues being encountered by learners in this area. If faculty members are adamant that understanding and teaching advocacy is a challenge but improving instruction in this field is not a priority it creates a real problem for education surrounding the Health Advocate role.

### Barriers to health advocacy education

The literature suggests that residents feel that health advocacy is important and merits training but that current educational practices may not suffice.^17^–^19^,^20^ A small number of barriers to adequate advocacy training have been identified via current literature.

Constrained time, both overall and protected learning time, was commonly noted as a barrier, as was the overall level of resident stress.^7^,^18^,^24^–^26^ Lack of clarity within the Health Advocate role was noted in two studies,^7^,^18^ with one reporting that none of the participants knew the RCPSC definition of health advocacy. Furthermore, from a teaching perspective, the lack of a specific curriculum for health advocacy training and the lack of a gold standard for assessment were also noted to be barriers to quality education.^7^,^26^

One study from a Canadian university used focus groups including both residents and faculty, from various specialties, to yield a number of potential barriers that may be more broadly applicable.^7^ In an ideological sense, both learners and faculty bemoaned health advocacy as unpaid charitable work, something that may detract from its relative value among other physician roles. There was also a notable discordance between the residents’ accounts of their preceptors’ limited engagement in health advocacy and the faculty members’ beliefs that it was a large part of their practice. This is an important finding as role modeling is heavily relied upon for health advocacy education.^7^,^18^ This implies that teachers need to be more explicit (and perhaps honest) about their advocacy activities. A more recent paper with the same authors echoed these findings, indicating that role modeling, although likely the most popular form of teaching, is insufficient in isolation.^15^

A review of Program Director needs at the University of Ottawa found a number of barriers to effective assessment of all non-Expert CanMEDS roles, with assessment of the Health Advocate role being seen as the most challenging. The barriers which limited quality assessment were low perceived value of the non-Expert roles, particularly given the high value placed on the Medical Expert role during the RCPSC exams, lack of incentive to assess proficiently, and the lack of clear benchmarks for resident ability at various stages of training.^26^

### Current practice

Not surprisingly, given educator frustration with the role and lack of health advocacy literature overall, little has been written regarding current teaching and assessment practices in Canada. With respect to teaching, role modeling appears to the most commonly used method,^15^ although only select faculties have explicitly published articles regarding curricula.^15^,^27^,^28^ One institution designed a health advocacy module that was implemented into the curriculum using an identified champion for the role, increased explicitness at grand rounds, initiation of resident projects and the introduction of a health advocacy day.^27^ Nationally, pediatric emergency fellows designed a conference to address their specific needs for education within all the non-Expert CanMEDS roles.^28^ Flynn and colleagues designed an operational curriculum framework for training in health advocacy designed to prepare for six attributes deemed necessary for a health advocate: altruistic, assertive, up-to-date, honest, knowledgeable, resourceful.^15^ A number of teaching methods for the framework were presented including role modeling, large group learning and small group cases, potentially creating a platform for different specialties to design an explicit curriculum for use.

With respect to assessment, the RCPSC has published *The CanMEDS Assessment Tools Handbook*,^29^ providing six preferred tools for assessing health advocacy. The tools include: essays, short-answer questions, in-training evaluation reports (ITERs), objective structured clinical evaluations (OSCEs), multi-source feedback, and portfolios. Chou and colleagues conducted a national survey of Program Directors looking to see which tools are actually in use to assess the seven CanMEDS roles.^30^ They showed that the vast majority of programs use ITERs to assess the Health Advocate role, with oral examination, OSCEs, and short answer questions also used by over 10% of respondents. The survey found assessment of the Health Advocate role garnered the lowest amount of satisfaction, aligning with findings of previously stated research. Despite the majority of programs using ITERs and the tools provided by the RCPSC, there does not seem to be a gold (or even a bronze) standard for assessment, which is reflected in the decreased level of satisfaction surrounding the role among educators.^24^

### Future directions

There appears to be no sign that health advocacy will lose its official status among the core roles of medicine. With the 2015 iteration of the CanMEDS roles being introduced, some adjustments to the role of Health Advocate are taking place. The role is being simplified from four to two key competencies, isolating advocacy at both the direct patient level and the population/community level, with only three “enabling competencies” within each key competency.^11^ There will be an increased emphasis on continuous quality improvement under the health advocacy umbrella. Importantly, the language has been altered to promote advocacy as an action performed *with* patients and *with* community groups as opposed to *for* them, in general. This represents a paradigm shift towards the communal sense of advocacy with patients, away from the more paternalistic approach of advocating for patients.^6^,^11^ The changes will perhaps help to address some of the confusion among front-line educators about the role itself and address perceptions of health advocacy as seemingly overwhelming. Data on the subject will take time to accrue.

There is also a growing movement in medical education in North America towards the increased application of novel concepts to describe and quantify student progress and level of responsibility. Two such concepts are milestones, which stage resident development within competencies, and entrustable professional activities (EPAs), which describe the level of supervision required for active tasks.^31^,^32^ As these concepts gain further acceptance moving forward, students and educators will need to be comfortable identifying the precise tasks which fall within the realm of health advocacy and be able to assess and promote step-wise progression in the learner’s abilities. Perhaps the more simplistic nature of the “enabling competencies” within the CanMEDS 2015 framework will be helpful in this regard.

## Conclusions

Health advocacy is of paramount importance to clinical practice and is an official pillar of current medical education at a variety of levels. The ability to advocate for and with patients, particularly those who are in exceedingly vulnerable positions, is a critical skill for physicians to possess. Yet, there is a worrisome lack of value ascribed to health advocacy as compared to other CanMEDS roles. Furthermore students feel inadequately taught and educators describe advocacy as very challenging to teach and assess. Key barriers limiting proficient education on the subject include limited resources and published literature on the topic for educators, lack of a gold standard for assessment, lack of clarity within the role, and insufficient explicit role modeling in practice. Increasing published literature and awareness of the role in practice could improve the quality of health advocacy education, particularly as the 2015 CanMEDS framework is being worked into practice. Individual specialty bodies can act at the forefront of this movement and it is paramount that physicians working in emergency departments, with children and with other vulnerable patient groups, be involved. Rotations through pediatric emergency medicine and similar services could provide a platform to discuss and highlight the importance of advocacy with the potential to foster a generation of residents in many disciplines who are competent in advocacy skills and see it as more than just an impractical ideal.
